# Cytotoxic and Antibacterial Angucycline- and Prodigiosin- Analogues from the Deep-Sea Derived *Streptomyces* sp. SCSIO 11594

**DOI:** 10.3390/md13031304

**Published:** 2015-03-16

**Authors:** Yongxiang Song, Guangfu Liu, Jie Li, Hongbo Huang, Xing Zhang, Hua Zhang, Jianhua Ju

**Affiliations:** 1 CAS Key Laboratory of Tropical Marine Bio-resources and Ecology, Guangdong Key Laboratory of Marine Materia Medica, RNAM Center for Marine Microbiology, South China Sea Institute of Oceanology, Chinese Academy of Sciences, 164 West Xingang Road, Guangzhou 510301, China; E-Mails: songx@scsio.ac.cn (Y.S.); lijietaren@yeah.net (J.L.); huanghb@scsio.ac.cn (H.H.); beishidazhangxing@163.com (X.Z.); 2 Department of Clinical Biochemistry, Institute of Clinical Laboratory Medicine, Guangdong Provincial Key Laboratory of Medical Molecular Diagnostics, Guangdong Medical College, No. 1 Xincheng Road, Dongguan 523808, China; E-Mail: 454143108@qq.com

**Keywords:** cytotoxicity, antibacterial, marangucyclines, deep-sea, *Streptomyces* sp. SCSIO 11594

## Abstract

Two new C-glycoside angucyclines, marangucycline A (**1**) and marangucycline B (**2**), along with three known compounds, dehydroxyaquayamycin (**3**), undecylprodigiosin (**4**) and metacycloprodigiosin (**5**), have been identified as products of the deep-sea sediment strain *Streptomyces* sp. SCSIO 11594. New structures were elucidated on the basis of HRESIMS, 1D and 2D NMR analyses and comparisons to previously reported datasets. Compounds **2** and **4** displayed *in vitro* cytotoxicity against four cancer cell lines A594, CNE2, HepG2, MCF-7 superior to those obtained with cisplatin, the positive control. Notably, compound **2** bearing a keto-sugar displayed significant cytotoxicity against cancer cell lines with IC_50_ values ranging from 0.24 to 0.56 μM; An IC_50_ value of 3.67 μM was found when using non-cancerous hepatic cell line HL7702, demonstrating the cancer cell selectivity of **2**. Compounds **1**–**3** were proved to have weak antibacterial activities against *Enterococcus faecalis* ATCC29212 with an MIC value of 64.0 μg/mL. Moreover, **3** displayed selective antibacterial activity against methicillin-resistant *Staphylococcus epidermidis* shhs-E1 with an MIC value of 16.0 μg/mL.

## 1. Introduction

Reports of increasing incidences of various cancers and the rise of multidrug resistant bacteria have inspired and renewed interest in the discovery of new secondary metabolites from marine-derived microorganisms as new drugs or new drug leads [1,2,3]. Deep-sea derived microorganisms, by virtue of their extreme living environments and selective pressures to which they have adapted, are considered especially exciting as potentially rich sources of new agents for drug discovery [4,5]. Historically speaking, terrestrial actinomycetes have been instrumental in the discovery of important secondary metabolites including antibiotics, antitumor agents, immunosuppressive agents and enzyme inhibitors [6]. This role of “discovery catalyst” is now gradually shifting to marine-derived actinomyces due to diminishing rates of new compound discovery and increasingly frequent “rediscovery” of known agents from terrestrial actinomyces [7,8,9]. Our continuing studies of cytotoxic and antibacterial compounds from marine-derived actinomyces, especially from the deep-sea derived strains, have led to the discoveries of antibacterial and cytotoxic cyclic peptide marthiapeptide A [10], antimalarial marinacarbolines and indolactam alkaloids [11] from deep-sea derived *Marinactinospora thermotolerans* SCSIO 00652, cytotoxic and antibacterial marfuraquinocins and phenaziterpenes from a deep-sea sediment actinomycete *Streptomyces niveus* SCSIO 3406, antibacterial cyclic peptides desotamides and marformycins from the deep-sea sediment actinomycetes *Streptomyces scopuliridis* SCSIO ZJ46 [12] and *Streptomyces drozdowiczii* SCSIO 10141 [13], respectively.

**Figure 1:**
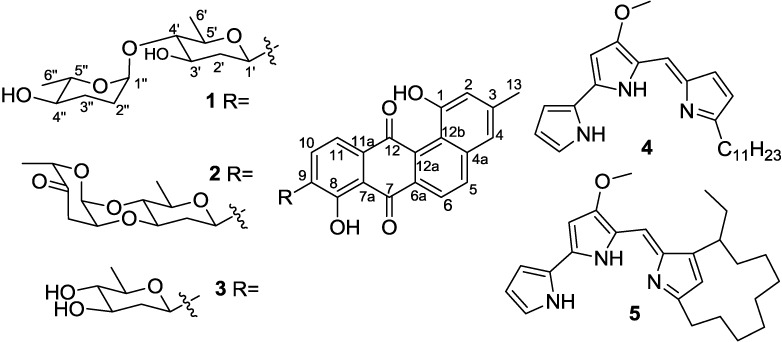
Secondary metabolites **1**–**5** from *Streptomyces* sp. SCSIO 11594.

In expanding our efforts to identify cytotoxic and antibacterial secondary metabolites from deep-sea derived actinomycetes, we isolated and identified *Streptomycetes* sp. strain SCSIO 11594 from a South China Sea sediment at a depth of 2403 m. This strain was found to produce cytotoxic and antibacterial substances warranting more detailed evaluation of these bioactivities. Metabolite analyses and subsequent structure elucidation efforts revealed two new C-glycoside angucyclines, marangucyclines A (**1**) and B (**2**), along with three known compounds identified as dehydroxyaquayamycin (**3**), undecylprodigiosin (**4**) and metacycloprodigiosin (**5**), as shown in Figure 1. Herein, we report the isolation, structure elucidation and bioactivity data for **1**–**5** from *Streptomyces* sp. SCSIO 11594.

## 2. Results and Discussion

### 2.1. Structure Elucidation

**Figure 2:**
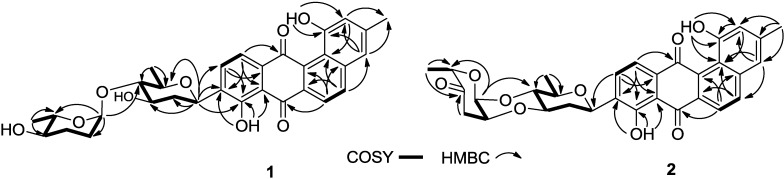
COSY and selected HMBC correlations for compounds **1** and **2**.

**Figure 3:**
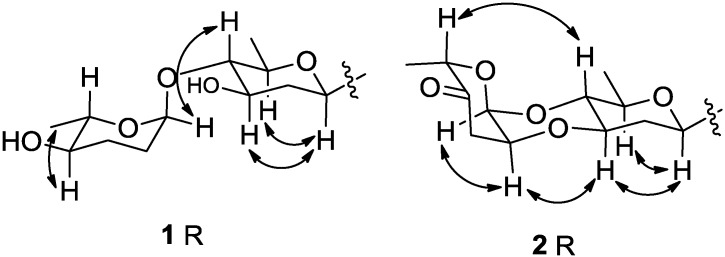
Selected NOESY correlations for the disaccharide moiety (*R*) of compounds **1** and **2**.

Compound **1**, marangucycline A, was isolated as a brown amorphous powder. The UV spectrum showed maxima at 239, 323 and 437 nm, indicating the presence of a large conjugated moiety. The IR spectrum showed characteristic absorptions at 3418 (hydroxyl), 2928 and 2877 (methyl) and 1632 cm^−1^ (the chelated quinone carbonyl). The compound’s molecular formula, C_31_H_32_O_9_, was determined on the basis of the HRESIMS peak at *m/z* 571.1946 [M + Na]^+^, requiring 16 degrees of unsaturation. Comprehensive analysis of the ^1^H and ^13^C NMR spectroscopic data strongly implied the presence of a typical angucycline of tetrangulol skeleton, with 1,8-dihydroxy-3-methyl-substituted and 9-C-glycosylated [14,15,16,17]. Detailed analysis of 1D (^1^H and ^13^C) and 2D (COSY, HMQC, HMBC, NOESY) NMR spectra of **1** (Supplementary Information), allowed the complete assignment of spectra signals (Table 1) and elucidation of the structure (Figure 2 and Figure 3).

**Table 1:** ^1^H (500 MHz) and ^13^C NMR (125 MHz) spectroscopic data of compounds **1** and **2** in CDCl_3_.

pos.	Marangucycline A (1)		Marangucycline B (2)	
	δ_C_	δ_H*,*_ mult. (*J* in Hz)	δ_C_	δ_H*,*_ mult. (*J* in Hz)
1	155.6, C		155.3, C	
2	120.2, CH	7.12, s	120.2, CH	7.15, s
3	142.1, C		142.1, C	
4	121.5, CH	7.23, s	121.3, CH	7.27, s
4a	132.6, C		132.5, C	
5	137.7, CH	8.11, d, *J* = 8.5	137.6, CH	8.14, d, *J* = 8.5
6	122.0, CH	8.29, d, *J* = 8.5	121.8, CH	8.32, d, *J* = 8.5
6a	135.0, C		134.8, C	
7	188.3, C		188.3, C	
7a	114.2, C		114.1, C	
8	158.2, C		157.8, C	
9	138.6, C		137.7, C	
10	133.6, CH	7.90, d, *J* = 8.0	133.6, CH	7.92, d, *J* = 8.0
11	121.3, CH	7.86, d, *J* = 8.0	121.2, CH	7.88, d, *J* = 8.0
11a	133.6, C		133.5, C	
12	189.6, C		189.4, C	
12a	139.3, C		139.2, C	
12b	120.2, C		120.1, C	
13	21.4, CH_3_	2.48, s	21.3, CH_3_	2.50, s
1-OH		11.43, br s		11.38, br s
8-OH		12.62, br s		12.66, br s
1′	71.3, CH	4.90, d, *J* = 11.2	71.5, CH	5.01, d, *J* = 11.0
2′	38.8, CH_2_	2.57, m; 1.46, m	36.6, CH_2_	2.48, m; 1.54, m
3′	71.5, CH	3.87, m	77.1, CH	3.84, ddd, *J* = 11.5, 9.0, 4.5
4′	89.2, CH	3.07, t, *J* = 6.5	74.5, CH	3.52, t, *J* = 9.0
5′	74.7, CH	3.57, m	74.6, CH	3.59, m
6′	18.6, CH_3_	1.38, d, *J* = 6.0	17.5, CH_3_	1.43, d, *J* = 6.0
1″	98.9, CH	4.92, br s	91.4, CH	5.19, d, *J* = 3.0
2″	27.3, CH_2_	1.93, m; 1.83, m	71.1, CH	4.35, q, 3.0
3″	30.1, CH_2_	1.87, m; 1.25, m	39.9, CH_2_	2.65, m
4″	71.8, CH	3.36, td, *J* = 10.0, 4.0	207.7, C	
5″	71.7, CH	3.91, m	77.8, CH	4.75, q, *J* = 6.5
6″	18.0, CH_3_	1.33, d, *J* = 6.0	16.2, CH_3_	1.39, d, *J* = 6.5

^1^H NMR spectra revealed the presence of two intramolecularly H-bonded hydroxyl groups at δ_H_ 12.62 and 11.43. Two pairs of *ortho*-coupled aromatic proton signals appeared at δ_H_ 8.29 (d, *J* = 8.5 Hz) and δ_H_ 8.11 (d, *J* = 8.5 Hz), δ_H_ 7.90 (d, *J* = 8.5 Hz) and δ_H_ 7.86 (d, *J* = 8.5 Hz), which also were found at C-6 (δ_C_ 122.0) and C-5 (δ_C_ 137.7), C-10 (δ_C_ 133.6) and C-11 (δ_C_ 121.3) based on HSQC data. Another two singlet proton signals at δ_H_ 7.23 (s) and δ_H_ 7.12 (s) were associated with C-4 (δ_C_ 121.5) and C-2 (δ_C_ 120.2), respectively. HMBC correlations from δ_H_ 11.43 to C-1 (δ_C_ 155.6) and C-2, and from δ_H_ 12.62 to C-8 (δ_C_ 158.2), C-7a (δ_C_ 114.2) and C-9 (δ_C_ 138.6), enabled assignment of these two hydroxyl groups at C-1 and C-8, respectively. The singlet aromatic methyl (δ_H_ 2.48, δ_C_ 21.4) was confirmed at C-3 (δ_C_ 142.1) by HMBC correlations from δ_H_ 2.48 to C-3, C-2 and C-4. The tetrangulol skeleton was further elucidated by HBMC correlations of two pairs of *ortho*-coupled protons at C-5, C-6, C-10 and C-11 (Figure 2). Moreover, the ^1^H NMR spectra displayed seven proton signals between δ_H_ 4.92 and δ_H_ 3.07, consistent with seven ^13^C signals of sp^3^ methines between δ_C_ 98.9 and 71.3. These data support the presence of a disaccharide moiety composed of β-d-olivose and α-l-amicetose on the basis of 2D NMR correlation analyses (Figure 2 and Figure 3), and comparisons with previously reported data [18,19]. COSY correlations of H-1′ (δ_H_ 4.90)/H-2′b (δ_H_ 1.46), H-2′a (δ_H_ 2.57)/H-3′ (δ_H_ 3.87)/H-4′ (δ_H_ 3.07)/H-5′ (δ_H_ 3.57)/H-6' revealed the fragment of C-1′/C-2′/C-3′/C-4′/C-5′/C-6′. The HMBC correlation of H-1′/C-5′ confirmed the existence of an olivose ring. The important HMBC correlations from the anomeric methine proton (CH-1′) to C-8, C-9, and C-10 indicated the C-glycosidic bond (C-9−C-1′) between the aglycone and olivose unit. Similarly, the COSY correlations of H-1″ (δ_H_ 4.92)/H-2″b (δ_H_ 1.83)/H-3″a (δ_H_ 1.87)/H-4″ (δ_H_ 3.36)/H-5″ (δ_H_ 3.91)/H-6″ (δ_H_ 1.33), and the HMBC correlation of H-1″/C-5″ disclosed the presence of a six membered deoxysugar, which was elucidated as amicetose by comparing the ^1^H and ^13^C NMR data with those reported [18]. Further HMBC correlations from H-1″ to C-4′ revealed the connection of C-1″-*O*-C-4′ between olivose and amicetose. The relative configurations of two sugar moieties were confirmed by NOESY correlations of H-1′/H-3′, H-1′/H-5′, H-1″/H-4′ and H-4″/CH_3_-6″ (Figure 3).

Compound **2**, named marangucycline B, was obtained as a brown amorphous powder. The IR spectrum showed one additional ketone absorption at 1730 cm^−1^ and the characteristic hydroxyl group absorption at ≈3400 cm^−1^ was almost completely absent. The molecular formula C_31_H_28_O_9_ was determined by HR-ESI-MS, which was four mass units less than that of compound **1**, indicating 18 degrees of unsaturation. Therefore, compound **2** was presumed to have one new ketone group and one additional ring relative to compound **1**. The ^13^C NMR spectrum were similar with those of **1** (Supplementary Information), except that one carbonyl signal at δ_C_ 207.7 (C-4″) was observed. Moreover, one of the anomeric carbone (C-1″) signal was changed from δ_C_ 98.9 in **1** to δ_C_ 91.4 in **2**. Comparing these characteristic data with the reported [19] revealed the presence of a cinerulose B unit in **2**, which subsequently proved by 2D NMR analyses (Figure 2). COSY spectrum indicated the fragments of C-1″/C-2″/C-3″ and C-5″/C-6″. The HMBC correlations of CH-1″/C-5″, CH_2_-3″/C-4″, and CH_3_-6″/C-5″ confirmed the structure elucidation of cinerulose B. The HMBC correlation from CH-1″ to C-4′ revealed the C-1″-*O*-C-4′ linkage. Meanwhile, the ^13^C NMR resonance of C-4′ shifted upfield from δ_C_ 89.2 in **1** to δ_C_ 74.5 in **2**, indicating the connection of C-2″-*O*-C-3′. Consequently, the disaccharide in **2** was determined to be a cinerulose B-(1→4, 2→3)-olivosyl unit. The NOESY correlations of H-1′/H-3′, H-1′/H-5′, H-3′/H-2″, H-2″/H-1″ and H-4′/H-5″ confirmed the relative configurations of the two sugars (Figure 3). The ^1^H and ^13^C NMR data for this disaccharide were consistent with these previously reported for compounds with α-cinerulose B-(1→4, 2→3)-β-olivosyl [19]. In a fashion analogous to that applied to compound **1**, the skeleton of **2** was determined by comprehensive analyses of COSY, HMQC and HMBC spectra (Figure 2 and Figure 3).

In addition to compounds **1** and **2**, *Streptomycetes* sp. strain SCSIO 11594 was found to produce known compounds dehydroxyaquayamycin (**3**) [14], undecylprodigiosin (**4**) and metacycloprodigiosin (**5**) [20]. The structures were determined by comparative analyses using previously reported MS, ^1^H, and ^13^C NMR data.

### 2.2. Cytotoxicities and Antibacterial Activities

The angucyclines are a large group of natural products; members are characterized by an angular tetracyclic (benz[α]anthracene) structure with a hydrolysable sugar moiety. Angucyclines often express a broad range of biological activities. Members of the angucyclines have been noted as potent cytotoxins, antibacterials, antivirals and as inhibitors of assorted enzymes and of platelet aggregation [17,21]. The first reported compounds of this class were tetrangomycin and tetrangulol [17]. To our knowledge, the sugar unit of these species was usually linked at the tetrangomycin C-9 with a C-C bond and at the tetrangulol C-8 with a C-O bond. The antitubercular and cytotoxic compound dehydroxyaquayamycin (**3**), was the first compound shown to have a C-9 linked sugar unit using C-C connectivity with the tetrangulol skeleton. This agent was obtained as a derivative of aquayamycin [15] and later isolated as a natural product from the marine-derived *Streptomyces* sp. BCC45596 [14]. Compounds **1** and **2** were additions to this class of natural products; **1**–**3** were isolated from marine-derived *Streptomyces* sp. SCSIO 11594 as secondary metabolites.

Compounds **1**–**5** were tested for potential *in vitro* cytotoxicity against human lung cancer cell line A549, human nasopharyngeal carcinoma cell line CNE2, human breast adenocarcinoma cell line MCF-7, human hepatocarcinoma cancer cell line HepG2, and the normal hepatic cell line HL7702. The results were shown in Table 2. These data indicate that compound **4** is ≈1–10-fold more cytotoxic than the positive control cisplatin whereas compound **2**, with its keto-sugar moiety, is ≈10–20-fold more potent than cisplatin. Interestingly, **2** and **4** demonstrate significant cytotoxic selectivity, with estimated therapeutic ratio values of 7–5 and 3–45, respectively, as reflected by comparisons of tumor cell-derived IC_50_ values and those obtained using HL7702 cells (non-cancerous control). Detailed examination of the structure-activity relationship (SAR) of the cytotoxicities of compounds **1**–**3** revealed that (i) the presence of ketone group and C-2″-*O*-C-3′ connection in the disaccharide moiety of compound **2** is critical important for the cytotoxicity; and (ii) the appearance of α-amicetose in the disaccharide chain sharply decreased the cytotoxicity. The SAR study of compounds **4** and **5** revealed that the open form of the aliphatic side chain plays an important role for the cytotoxicity. The anticancer activity of the related natural products has been described in a number of literatures [22,23,24], and the structure activity relationships of related synthetic natural products have been reported [25,26,27,28].

The antibacterial activities of compounds **1**–**5** were evaluated using MRSE shhs-E1, methicillin-resistant *Staphylococcus aureus* (MRSA) shhs-A1, *Staphylococcus aureus* ATCC 29213, *Enterococcus faecalis* ATCC29212, *Escherichia coli* ATCC 25922, *Micrococcus luteus*, and multidrug resistant *Pseudomonas aeruginosa*. These assays revealed that **1**–**3** have weak antibacterial activity against *Enterococcus faecalis* ATCC29212 with a MIC of 64.0 μg/mL in all cases and that **3** is selective against MRSE shhs-E1 demonstrating an MIC of 16.0 μg/mL. Compounds **4** and **5** did not show antibacterial activities against any of the above tested bacteria at a concentration up to 64.0 μg/mL.

**Table 2:** Summary of *in vitro* cytotoxicities (IC_50_ in μM) for **1**–**5** against four human cancer cell lines and one normal hepatic cell line HL7702 (*n =* 3) and estimated therapeutic ratio (TR) values.

Agent	A549	CNE2	MCF-7	HepG2	HL7702	TR
**1**	>50.0	>50.0	>50.0	>50.0	>50.0	-
**2**	0.45 ± 0.03	0.56 ± 0.02	0.24 ± 0.003	0.43 ± 0.05	3.67 ± 0.07	7–15
**3**	16.40 ± 0.19	22.27 ± 0.07	23.65 ± 0.09	18.81 ± 0.12	49.34 ± 0.17	2–3
**4**	0.85 ± 0.01	0.28 ± 0.02	1.11 ± 0.07	4.67 ± 0.09	12.47 ± 0.09	3–45
**5**	>50.0	>50.0	>50.0	>50.0	>50.0	-
Cisplatin	4.56 ± 0.04	3.75 ± 0.03	5.26 ± 0.07	4.14 ± 0.06	15.34 ± 0.08	3–4

### 2.3. Identification of Strain SCSIO 11594

**Figure 4:**
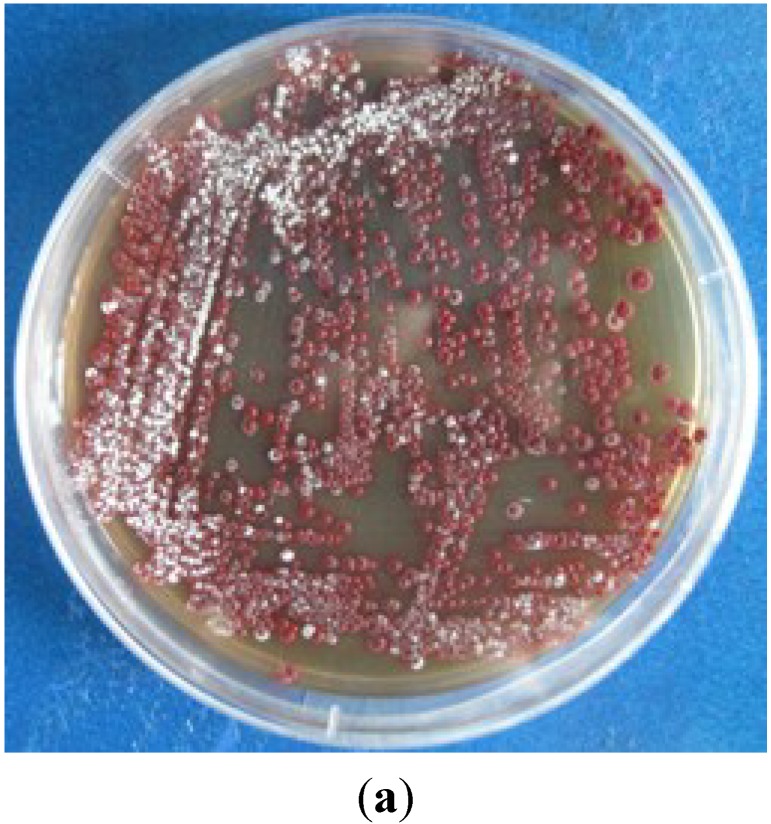

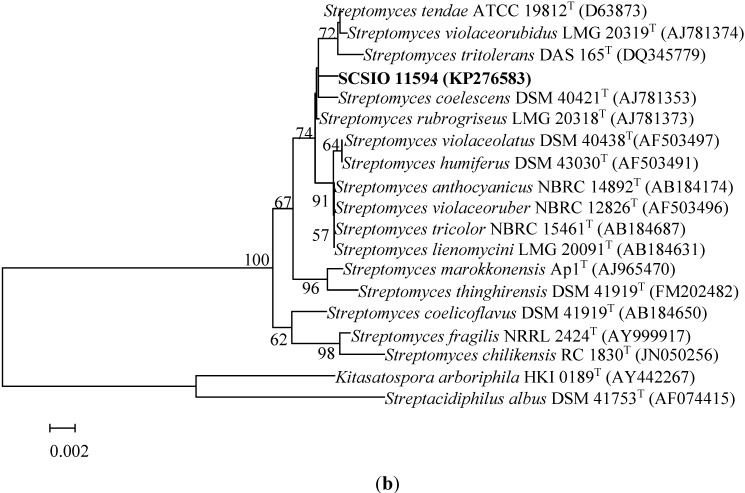
(**a**) Strain SCSIO 11594; (**b**) Neighbor-joining phylogenetic tree based on 16S rRNA gene sequences of strain SCSIO 11594 and members of the family *Streptomycetaceae*. Bootstrap values (expressed as percentages of 1000 replications) exceeding 50% are shown at the branch points.

Strain SCSIO 11594 (Figure 4a was isolated from a sediment sample collected from the South China Sea (115°27.751 E, 19°28.581 N) at a depth of 2403 m using HRA medium (histidine 0.1 g, raffinose 1.0 g, Na_2_HPO_4_ 0.3 g, KCl 1.7 g, MgSO_4_·7H_2_O 0.05 g, FeSO_4_·7H_2_O 0.01 g, CaCO_3_ 0.02 g, agar 12 g, pH7.2, seawater 500 mL, distilled water 500 mL) with incubation at 28 °C for up to 4 weeks. The strain is preserved at the RNAM Center for Marine Microbiology, South China Sea Institute of Oceanology, Chinese Academy of Sciences. Extraction of genomic DNA, PCR amplification, sequencing of the 16S rRNA gene, and phylogenetic analysis were performed as described by You and co-workers [29]. The 16S rRNA gene sequence has been deposited in GenBank with accession no. KP276583. The results of phylogenetic analyses showed that strain SCSIO 11594 should be a member of the genus *Streptomyces*. The 16S rRNA gene sequence of strain SCSIO 11594 demonstrated the highest similarity value to *Streptomyces rubrogriseus* LMG 20318^T^ (99.86%) and was found to cluster with members of the genus *Streptomyces* in the phylogenetic tree (Figure 4b).

## 3. Experimental Section

### 3.1. General Experimental Procedures

Optical rotations were determined using an MCP-500 polarimeter (Anton Paar, Graz, Austria). UV spectra were obtained with a UV-2600 spectrometer (Shimadzu, Tokyo, Japan). IR spectra were measured using an IRAffinity-1 spectrophotometer (Shimadzu, Tokyo, Japan). NMR spectra were acquired with an Avance 500 spectrometer (Bruker, Fällanden, Switzerland) at 500 MHz for the ^1^H nucleus and 125 MHz for the ^13^C nucleus. ESIMS and HRESIMS data were determined using an amaZon SL ion trap mass spectrometer and MaXis quadrupole-time-of-flight mass spectrometer (Bruker, Bremen, Germany), respectively. CC (Column chromatography) was performed on 100–200 mesh silica gel (Yantai Jiangyou Silica Gel Development Co., Ltd., Yantai, China). RP-MPLC (reversed phase-medium pressure preparative liquid chromatography) were carried out using the CHEETAH MP200 (Agela Technologies, Tianjin, China) and Claricep Flash columns filled with ODS (40−63 μm, YMC). RP-HPLC (high performance liquid chromatography) analyses were carried out using a Hitachi HPLC with YMC-Pack ODS-A column (250 × 10 mm, 5 μm).

### 3.2. Fermentation, Extraction and Isolation of the Compounds

The strain was inoculated to a modified ISP-4 agar plate (soluble starch 1.0%, K_2_HPO_4_ 0.1%, MgSO_4_·7H_2_O 0.1%, (NH_4_)_2_SO_4_ 0.2%, CaCO_3_ 0.2%, sea salt 3.0%, pH 7.2 before sterilization) from a glycerol tube under aseptic conditions and incubated 5 day at 28 °C. The mycelium was then transferred into 250 mL Erlenmeyer flasks each containing 50 mL of modified-AM2ab medium (soluble starch 0.5%, soybean powder 0.5%, yeast exact power 0.2%, bacterial peptone 0.2%, glucose 2.0%, KH_2_PO_4_ 0.05%, MgSO_4_·7H_2_O 0.05%, NaCl 0.40%, sea salt 3.0% CaCO_3_ 0.2%, pH 7.2 before sterilization) and incubated on rotary shakers with 200 rpm at 28 °C for 36 h. Each culture (seed) was then inoculated into 1 L Erlenmeyer flasks containing 200 mL of modified-AM2ab and fermentation continued under the conditions used to generate the seed cultures. Incubation was carried out under these conditions for 8 day. The 16 L of culture was then harvested and centrifuged at 3214× *g* (3900 rpm) for 10 min to yield the supernatant and mycelial cake, which was extensively extracted by butanone and acetone, respectively. The extracted residues were combined after HPLC analyses to validate extract contents.

The combined residues were subjected to silica gel CC using gradient elution initially using petroleum ether (P)/chloroform (C) (200/0, 100/100, v/v), and then with C/MeOH (M) (150/0, 147/3, 144/6, 141/9, 138/12, 145/15, 130/30, 75/75, v/v), to give ten fractions A1–A10. The fractions A4–A8 were combined and further subjected to ODS column MPLC by eluting with a linear gradient from 70/30/0.1 (MeCN/H_2_O/HAc) to 100/0/0.1 over 60 min at 15 mL/min with detection at 323 nm; elution was then continued with 100/0/0.1 for 30 min to ultimately afford fractions B1–B9. The fractions B6 and B7 and B8 and B9 were subjected to CC silica gel with a gradient elution (P/C 10/40, 0/50, C/M 49.5/0.5, 49/1, 48.5/1.5, v/v), respectively, to give fractions C1–17 and D1–17. The fractions C12 and C13 and D9–12 were repeatedly eluted by isocratic SP-HPLC elution with MeCN/H_2_O/HAc (95/5/0.1) at 2.5 mL/min and detection at 323 nm, respectively, to generate compound **1** (30.5 mg) at a retention time of 12.2 min and compound **2** (7.5 mg) with retention time 14.3 min. Similarly, compound **3** (3.7 mg) was isolated from B5 by repeated isocratic RP-HPLC elution with MeCN/H_2_O/HAc (85/15/0.1) and a retention time of 8.7 min. Fraction A3 was subjected to silica gel CC with gradient elution (P/C 50/0, 40/10, 30/20, 20/30, 10/40, 0/50, C/M 49.5/0.5, 49/1) to afford fractions E1–E8; E5–E7 were repeatedly subjected to preparative TLC (100% chloroform) to afford **4** (33.2 mg) and **5** (45.3 mg) with R_f_ values of 0.7 and 0.4, respectively.

***Marangucycline A*** *(**1**)*: brown amorphous powder; [α]^25^_D_ 35.0 (*c* 0.37, CHCl_3_); UV (CHCl_3_) λ_max_ (log ε) 239 (4.39), 323 (4.31), 437 (3.87) nm; IR (ATR) ν_max_ 3418, 2928, 2878, 1631, 1269, 1049 cm^−1^; ^1^H and ^13^C NMR spectroscopic data, Table 1; (−)-ESIMS *m/z* [M − H]^−^ 547.42, and (+)-HRESIMS *m/z* [M + Na]^+^ 571.1946, calcd for C_31_H_32_O_9_Na, 571.1939.

***Marangucycline B*** *(**2**)*: brown amorphous powder; [α]^25^_D_ 30.5 (*c* 0.19, CHCl_3_); UV (CHCl_3_) λ_max_ (log ε) 239 (4.15), 323 (4.03), 436 (3.58) nm; IR (ATR) ν_max_ 2922, 2852, 1730, 1635, 1254, 1109, 1069 cm^−1^; ^1^H and ^13^C NMR spectroscopic data, Table 1; (+)-HRESIMS *m/z* [M + H]^+^ 545.1796, calcd for C_31_H_29_O_9_, 545.1806; [M + Na]^+^ 567.1620, cacld for C_31_H_28_O_9_Na, 567.1626.

### 3.3. Cytotoxicity Assays

Compounds **1**–**5** were evaluated for cytotoxic activity using four human cancer cell lines, A549, CNE2, HepG2, MCF-7, one normal hepatic cell line HL7702 and previously reported MTT methodologies [30]. IC_50_ values were calculated using GraphPad Prism 5 software. All data were obtained in triplicate and are presented as means ± SD. Cisplatin was used as a positive control.

### 3.4. Antibacterial Activities Assay

The antibacterial activities of compounds **1**–**5** were assessed using seven strains of pathogenic bacteria including MRSE shhs-E1, MRSA shhs-A1, *Staphylococcus aureus* ATCC 29213, *Enterococcus faecalis* ATCC29212, *Escherichia coli* ATCC 25922, *Micrococcus luteus*, and multidrug resistant *Pseudomonas aeruginosa*. Dilution antimicrobial susceptibility tests for aerobic bacteria were carried out as previously reported [31,32]. Lowest concentrations of antimicrobial agents that completely inhibit cell growth in microdilution wells were determined by naked eye.

## 4. Conclusions

Five compounds including two new angucycline antibiotics, marangucycline A (**1**) and marangucycline B (**2**), were isolated from the deep-sea derived *Streptomyces* sp. SCSIO11594. Compounds **2** and **4**, especially the new C-glycoside angucycline compound **2**, displayed *in vitro* cytotoxicities against four cancer cell lines A594, CNE2, HepG2, MCF-7 superior to those noted with cisplatin. Remarkably, compounds **2** and **4** demonstrated significant anti-tumor selectivity. These data enable important correlations of structure to biological function and may be important for future drug lead efforts. Additionally, dehydroxyaquayamycin (**3**) was found to exert selective antibacterial activity against MRSE shhs-E1. This realization may prove important during the course of structure-activity relationship studies aimed at new antibacterial drug discovery/design studies.

## Supplementary Files

Supplementary File 1
