# Supplementary material for: Cytotoxic and Antibacterial Angucycline- and Prodigiosin- Analogues from the Deep-Sea Derived Streptomyces sp. SCSIO 11594
Source: Mar Drugs. 2015 Mar 16;13(3):1304–16. doi: 10.3390/md13031304 (PMC4377985; doi:10.3390/md13031304)
Supplement: Supplementary File 1 [file marinedrugs-13-01304-s001.pdf]

Supplementary Information

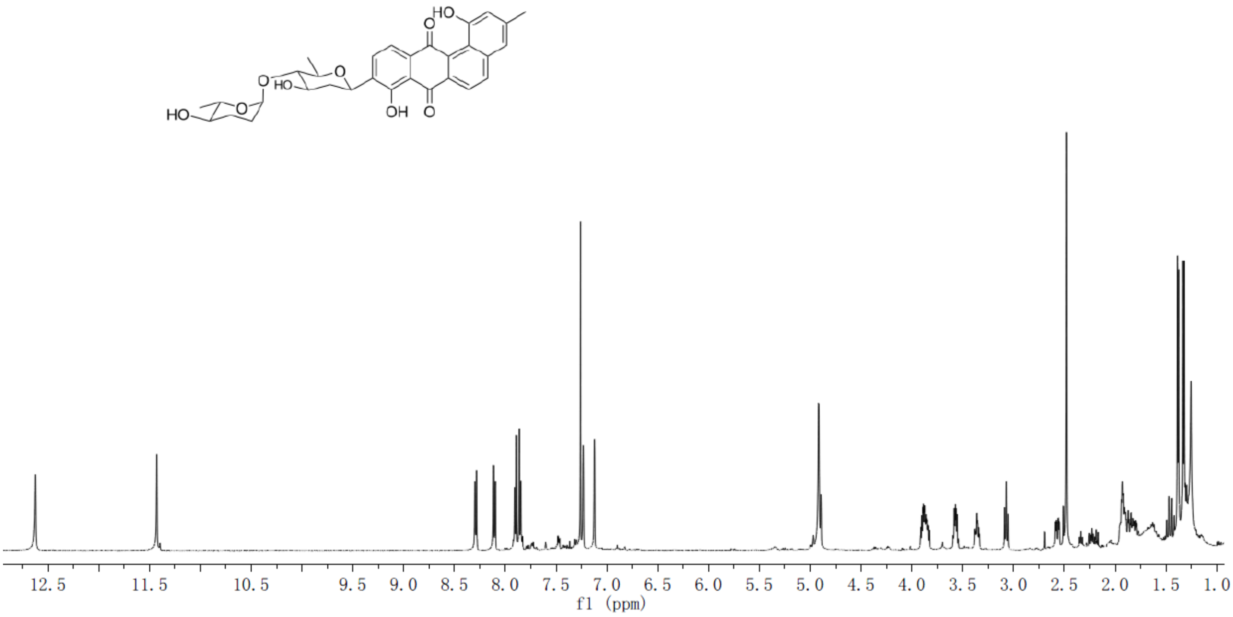

Figure S1. ^{1}H NMR (500 MHz) spectrum of **1** in $CDCl_3$.

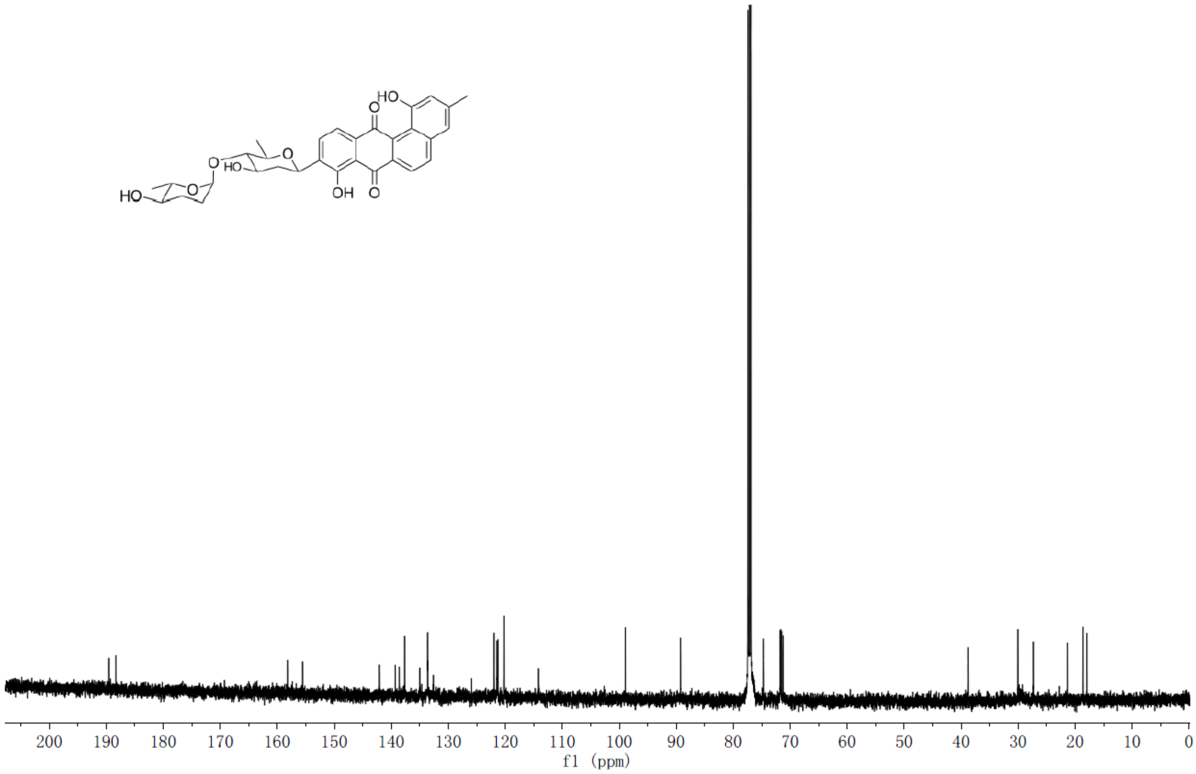

Figure S2. ^{13}C NMR (125 MHz) spectrum of **1** in $CDCl_3$.

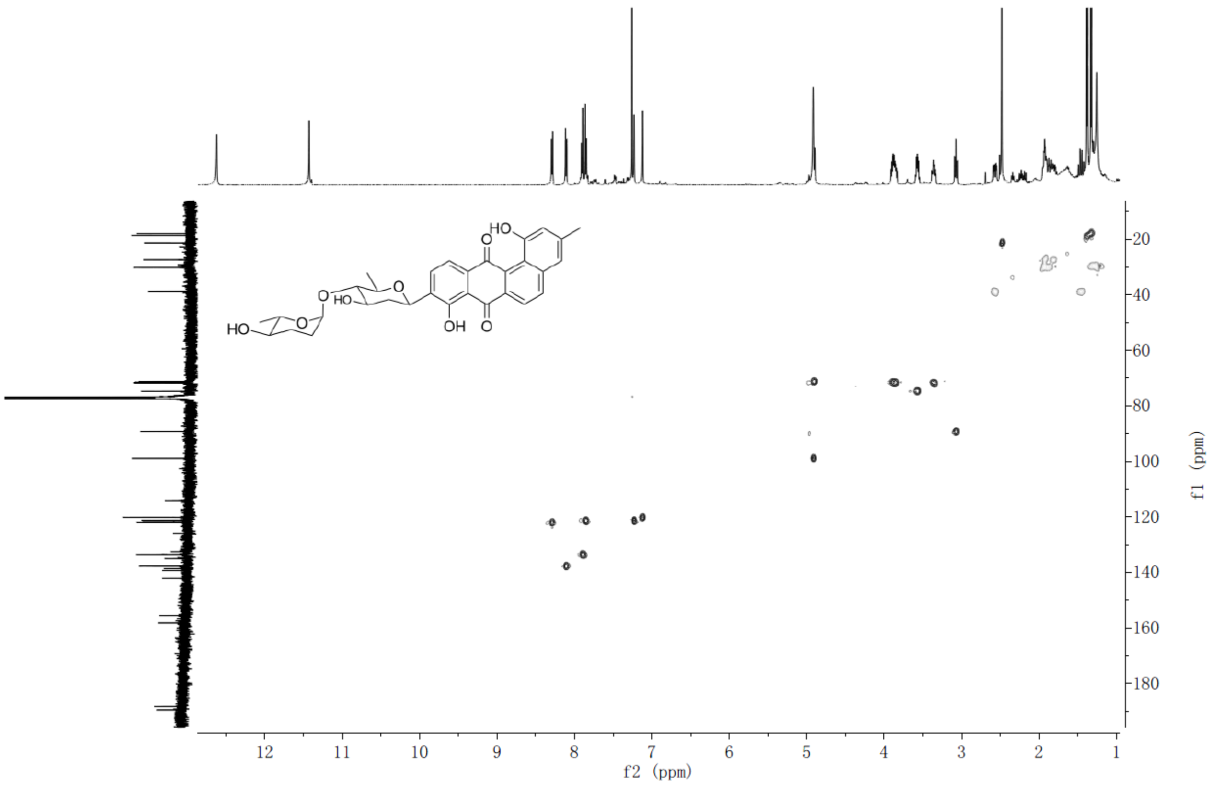

Figure S3. HMQC spectrum of **1** in $CDCl_3$.

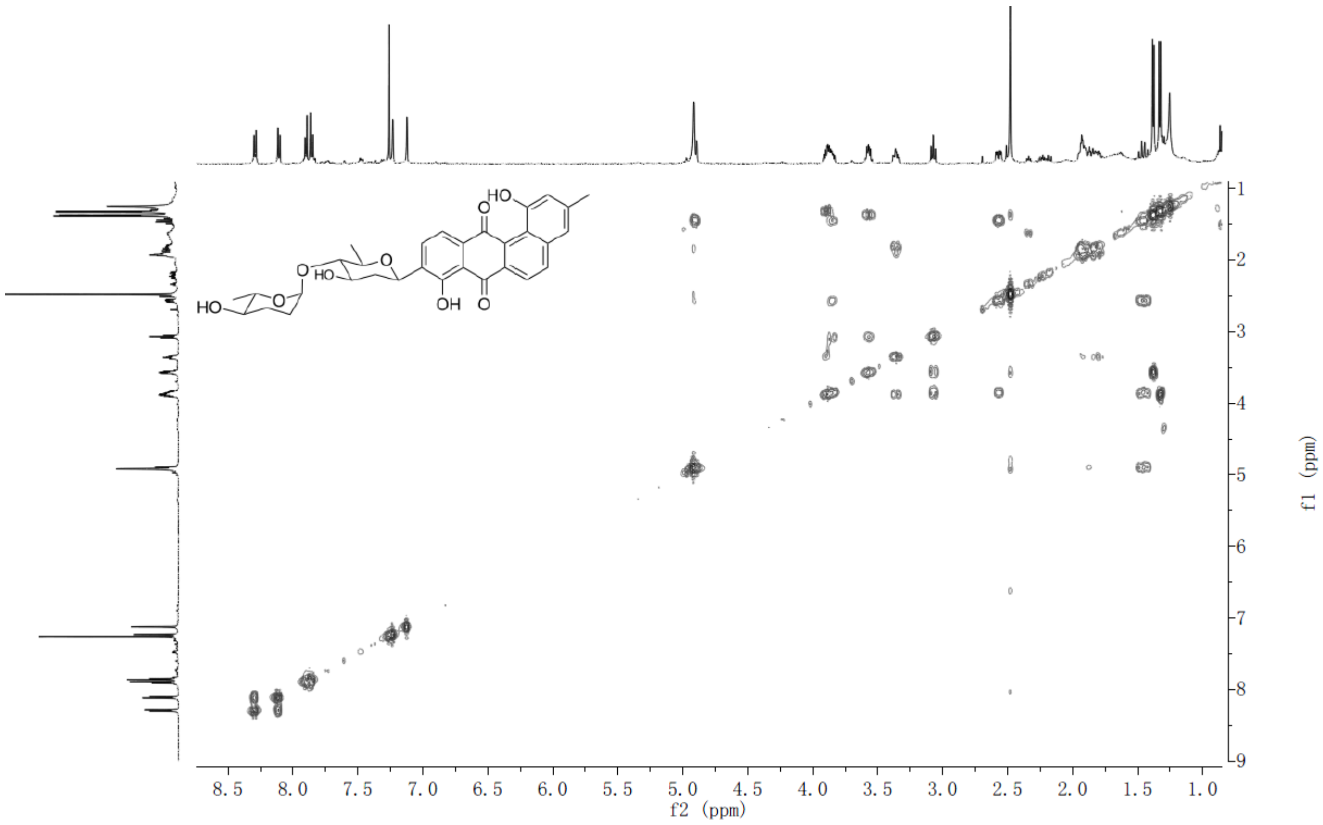

Figure S4. ^{1}H-^{1}H COSY spectrum of **1** in $CDCl_3$.

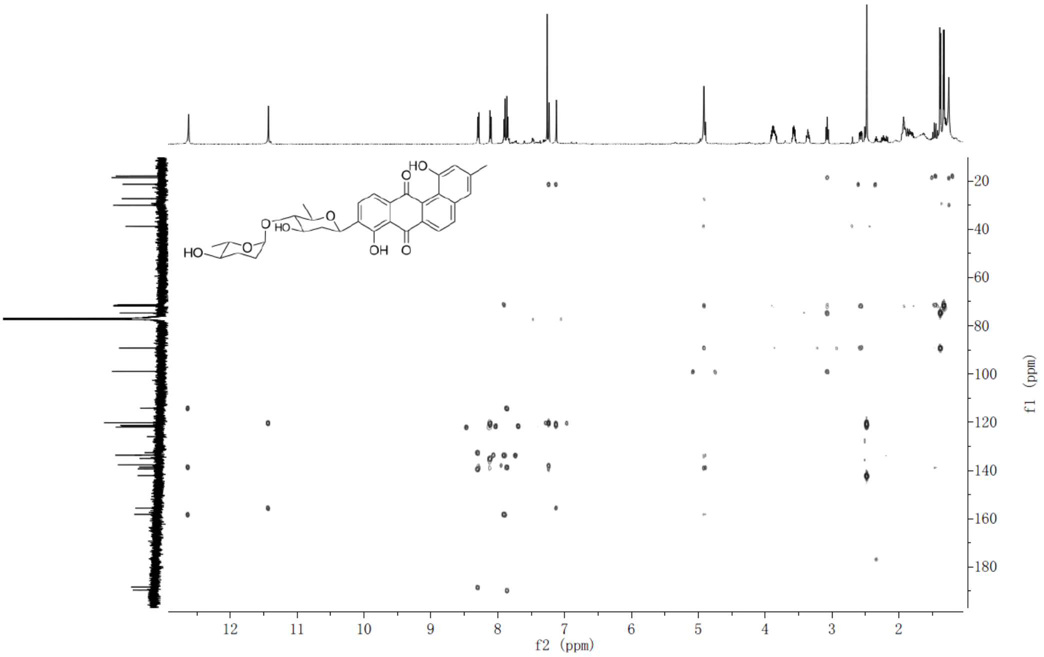

Figure S5. HMBC spectrum of **1** in $CDCl_3$.

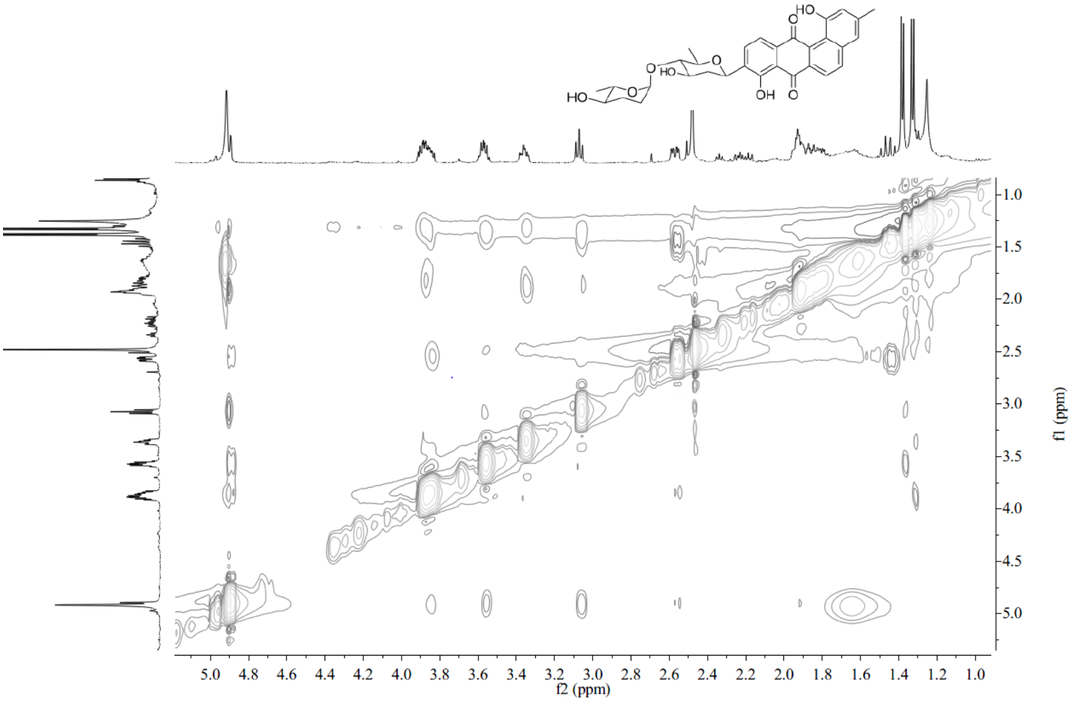

Figure S6. NOESY spectrum of **1** in $CDCl_3$.

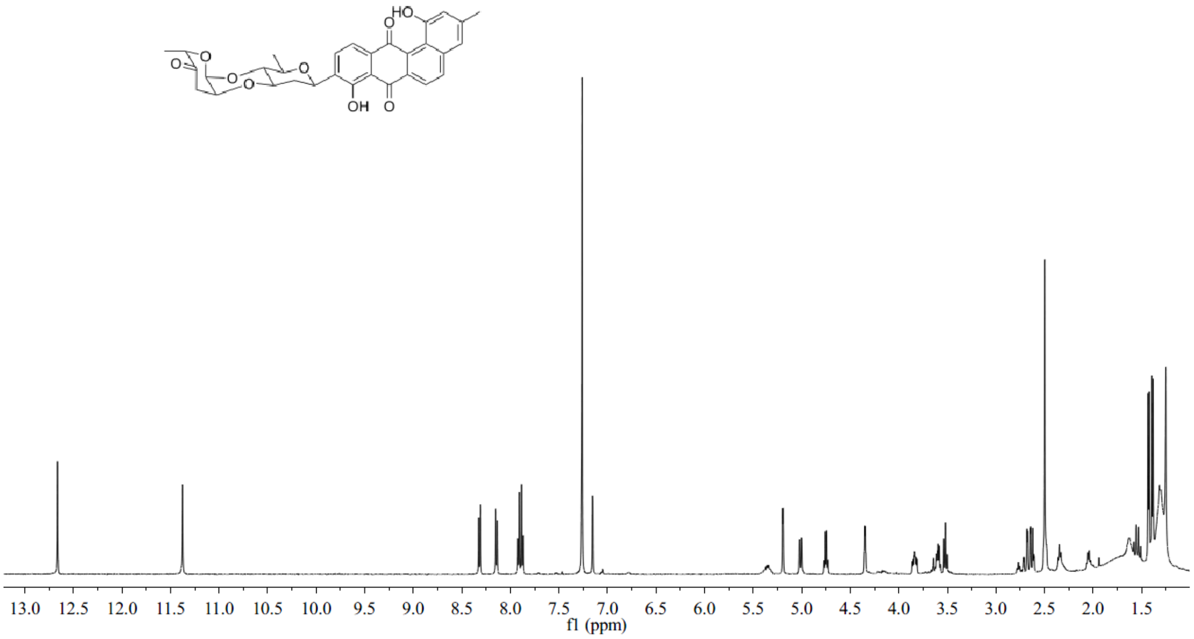

Figure S7. ^{1}H NMR (500 MHz) spectrum of **2** in $CDCl_3$.

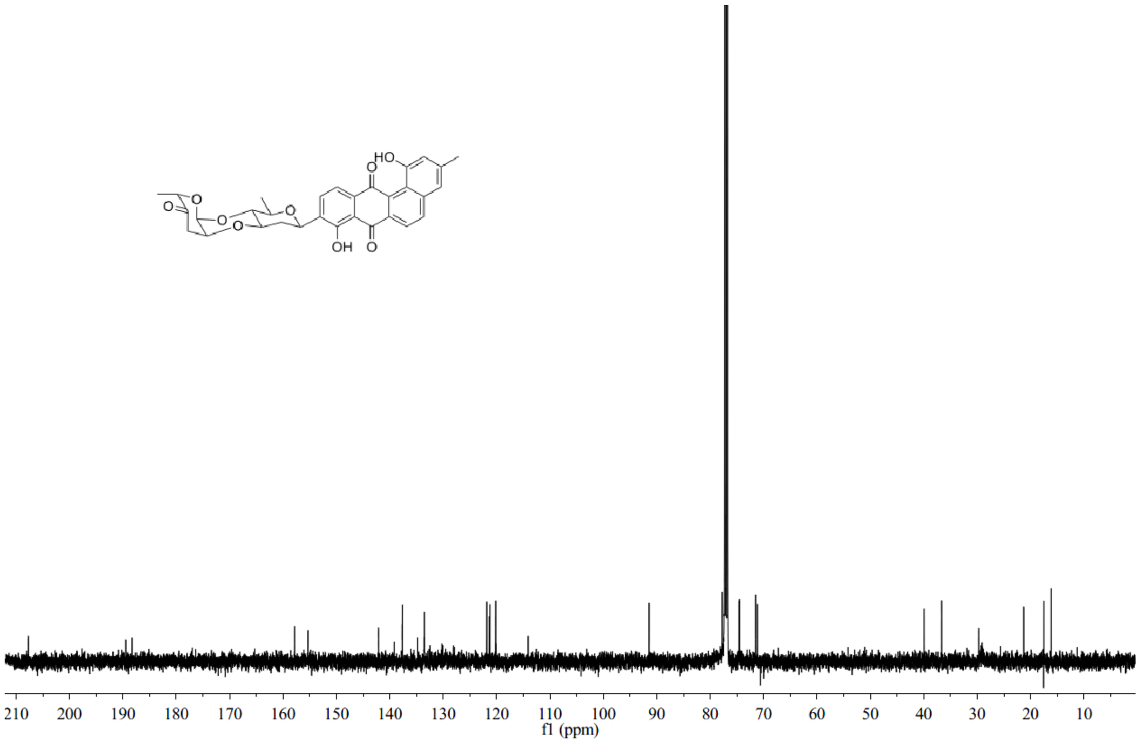

Figure S8. ^{13}C NMR (125 MHz) spectrum of **2** in $CDCl_3$.

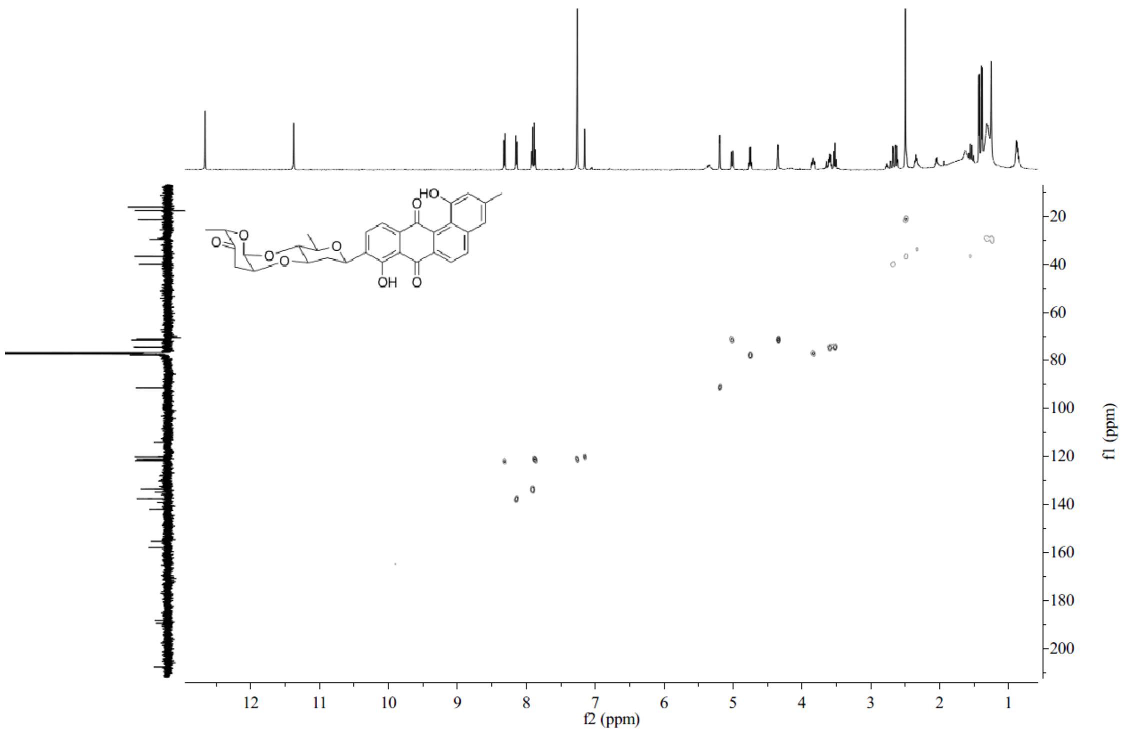

Figure S9. HMQC spectrum of **2** in $CDCl_3$.

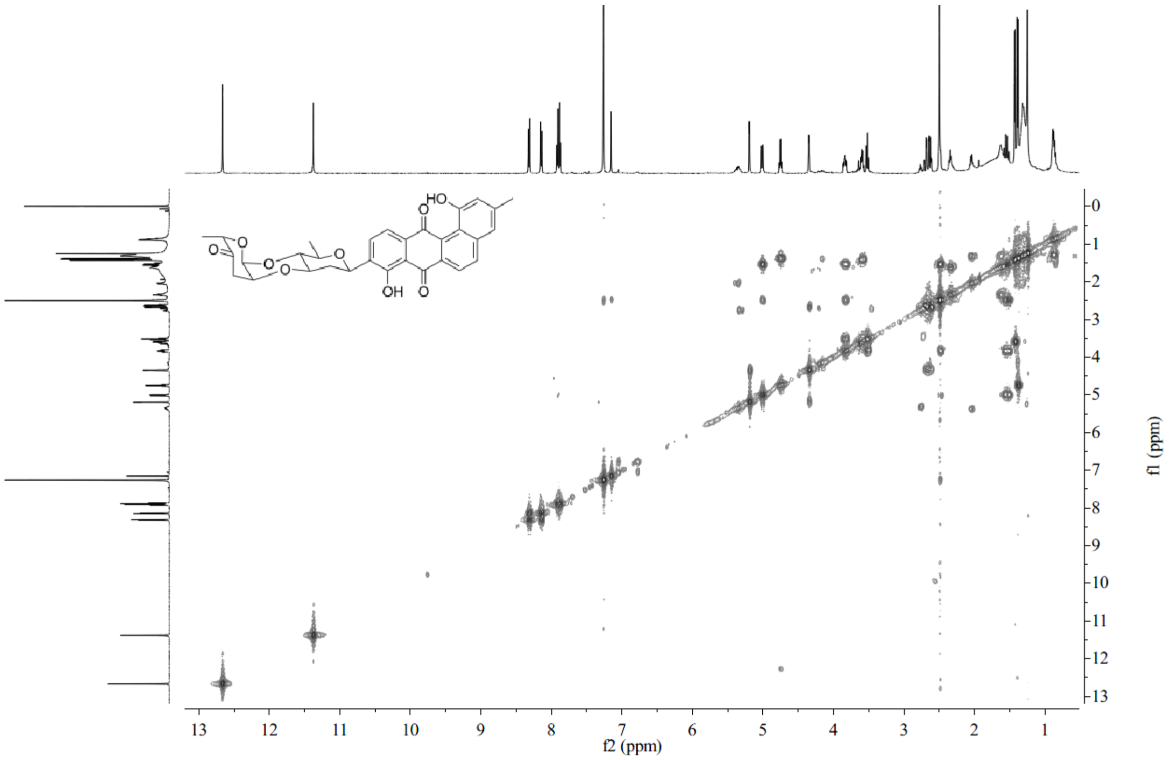

Figure S10. ^1H-^1H COSY spectrum of **2** in $CDCl_3$.

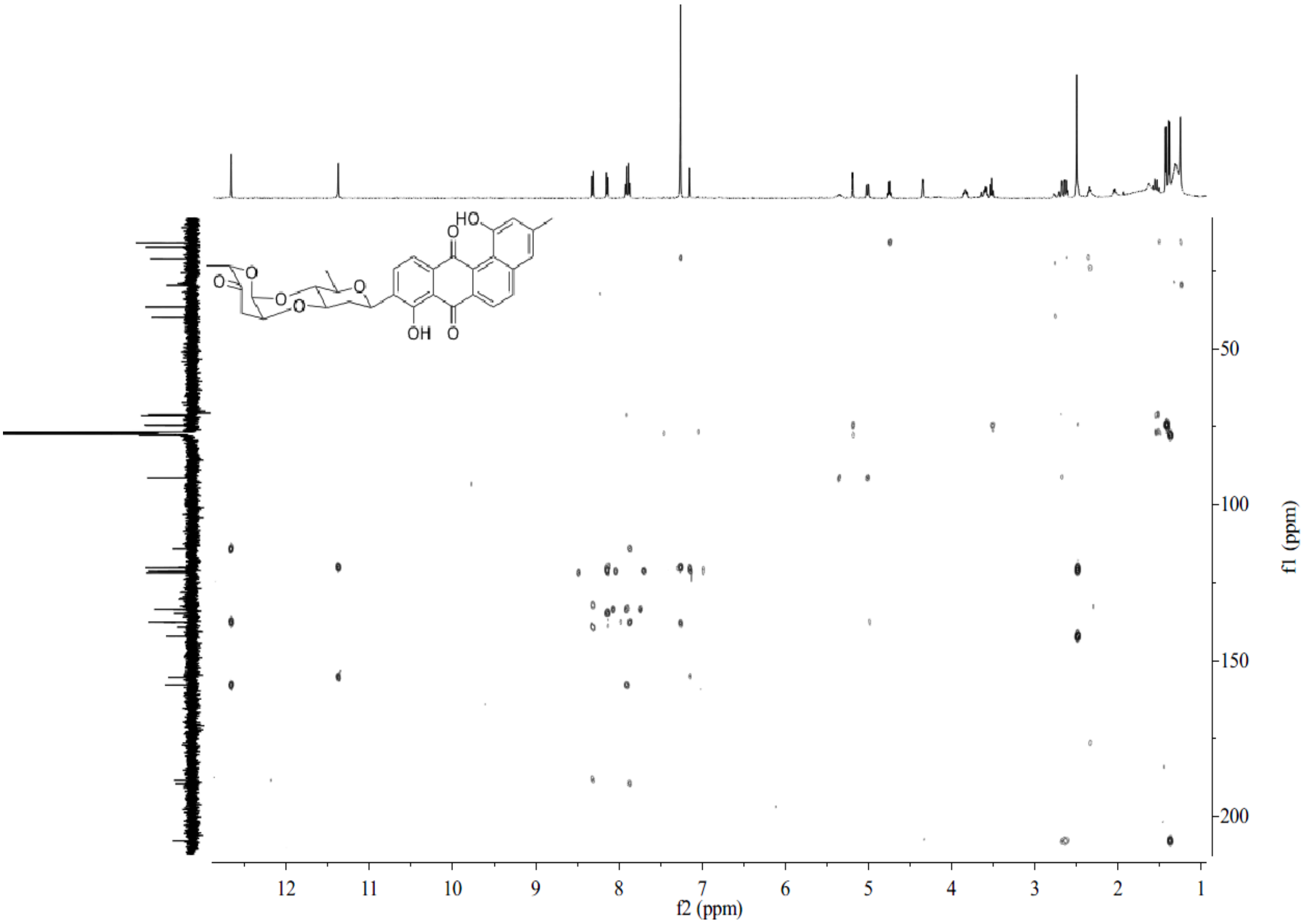

Figure S11. HMBC spectrum of **2** in $CDCl_3$.

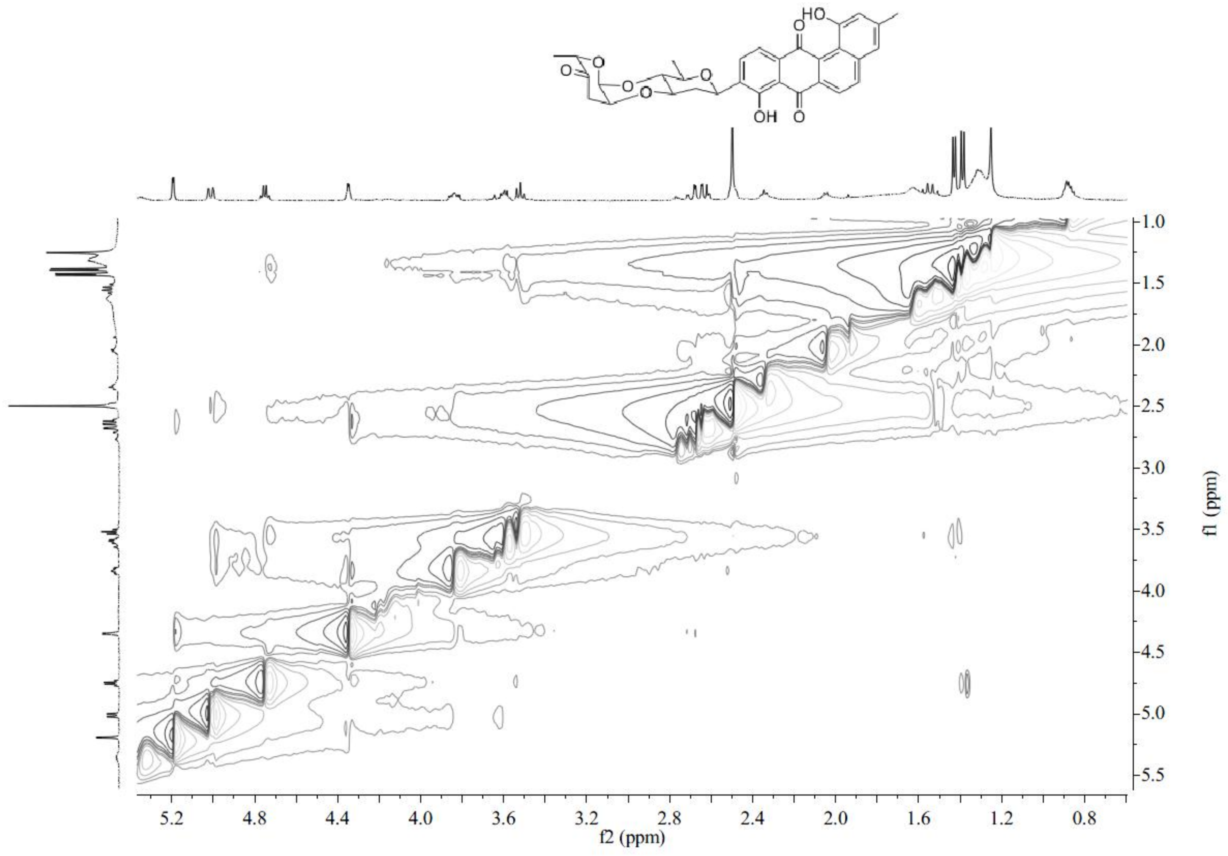

Figure S12. NOESY spectrum of **2** in $CDCl_3$.

© 2015 by the authors; licensee MDPI, Basel, Switzerland. This article is an open access article distributed under the terms and conditions of the Creative Commons Attribution license (http://creativecommons.org/licenses/by/4.0/).
